# Zero Fluoroscopy Radiofrequency Ablation of Paroxysmal Atrial Fibrillation and Epicardial Ventricular Tachycardia

**DOI:** 10.1016/j.jaccas.2025.105298

**Published:** 2025-10-08

**Authors:** Guang-an Liu, Bo Shao, Ruoxi Zhang, Binquan You, Feng Liu

**Affiliations:** Department of Cardiology, Suzhou Kowloon Hospital, Shanghai Jiao Tong University School of Medicine, Suzhou, China

**Keywords:** ablation, atrial fibrillation, ventricular tachycardia

## Abstract

**Background:**

Paroxysmal atrial fibrillation (AF) and epicardial ventricular tachycardia (VT) are complex arrhythmias requiring precise, safe ablation. Zero-fluoroscopy radiofrequency ablation (RFA) minimizes radiation exposure while maintaining procedural accuracy.

**FIH/Early Reports Summary:**

A 56-year-old male with diabetes, hypertrophic cardiomyopathy, AF, and VT underwent zero-fluoroscopy, single-stage RFA for both AF and epicardial VT. Integration of intracardiac echocardiography (ICE) and CARTO 3D mapping enabled effective ablation and restored sinus rhythm with symptom relief.

**Discussion:**

This case highlights the utility of ICE and CARTO 3D in enhancing procedural precision and safety. Eliminating fluoroscopy reduces radiation risks, particularly in complex cases.

**Novelty:**

This is the first report of single-stage, zero-fluoroscopy RFA for both AF and epicardial VT using ICE and 3D mapping.

## Graphical Abstract

**Figure:**
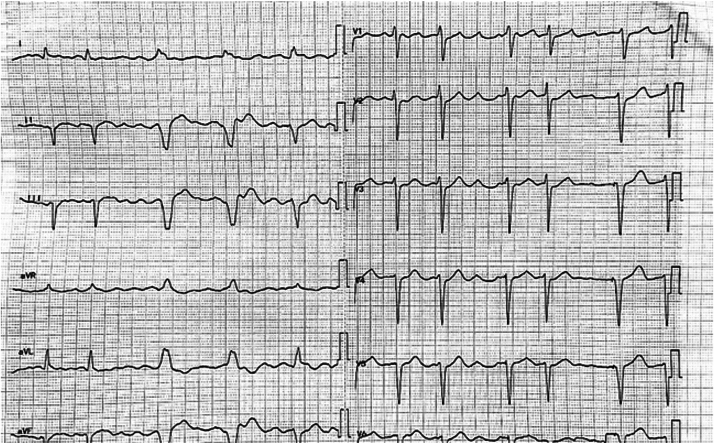

The integration of intracardiac echocardiography (ICE) with the CARTO three-dimensional (3D) mapping system has enabled significant advancements in the field of catheter ablation, particularly in complex arrhythmias such as atrial fibrillation (AF) and epicardial ventricular tachycardia (VT). This combined approach allows for precise and effective pericardiocentesis and ablation under zero fluoroscopy, mitigating the risks associated with radiation exposure. The integration of combining ICE with CARTO 3D mapping system enhances procedural accuracy and patient safety in the management of complex cardiac arrhythmias.

**Take-Home Messages**

• ICE and CARTO 3D enable precise, zero-fluoroscopy ablation of complex arrhythmias.
• This method improves safety and reduces radiation exposure for patients and clinicians.

## Past Medical History

A 56-year-old male patient with a well-controlled 4-year history of diabetes mellitus, but no hypertension, was diagnosed with hypertrophic cardiomyopathy, VT, and paroxysmal AF in 2017 following recurrent episodes of chest tightness, palpitations, and several instances of syncope. After undergoing radiofrequency ablation (RFA) for VT at another hospital, an implantable cardioverter-defibrillator was placed, and the patient was started on oral amiodarone therapy. This treatment resulted in relief from palpitations and chest tightness.

**Figure 1:**
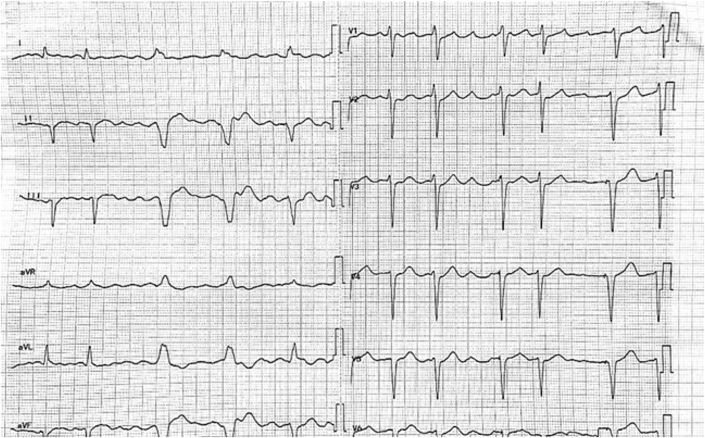
A Standard 12-Lead Surface Electrocardiogram Revealed Atrial Fibrillation

**Figure 2:**
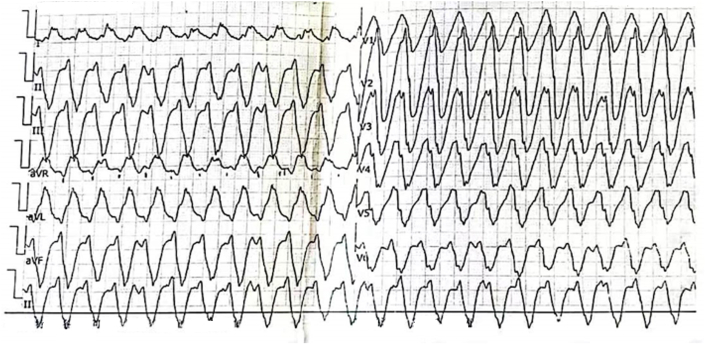
A Standard 12-Lead Surface Electrocardiogram Revealed Paroxysmal Ventricular Tachycardia

In 2019, the patient experienced intermittent episodes of palpitations and chest tightness, although the patient no longer experienced syncope. A standard 12-lead surface electrocardiogram (ECG) revealed AF (Figure 1). During episodes of palpitations, the ECG captured wide-QRS tachycardia, which was identified as paroxysmal VT (Figure 2). The implantable cardioverter-defibrillator recorded 38 VT events, triggering multiple episodes of antitachycardia pacing and cardioversion as part of its therapeutic intervention. However, in 2019, the patient did not undergo further RFA due to the relative stability of their condition and a shared decision to first optimize pharmacological management and antiarrhythmic therapy before considering invasive interventions. In addition, the patient showed partial response to antitachycardia pacing and cardioversion, which provided adequate symptom control, thus delaying the need for further ablation.

## History of Presentation

The patient was admitted to our hospital in 2022 and experienced frequent episodes of palpitations and chest tightness. Upon admission, laboratory tests showed normal levels of blood electrolytes, liver and kidney functions, and thyroid function. Echocardiography revealed a left atrial anterior-posterior diameter of 46 mm, a left ventricular diastolic diameter of 56 mm, and a left ventricular ejection fraction of 47%. Both the ventricular septum and left ventricular posterior wall thickness measured 13 mm. Esophageal echocardiography confirmed the absence of a left atrial appendage thrombus.

## Management

**Figure 3:**
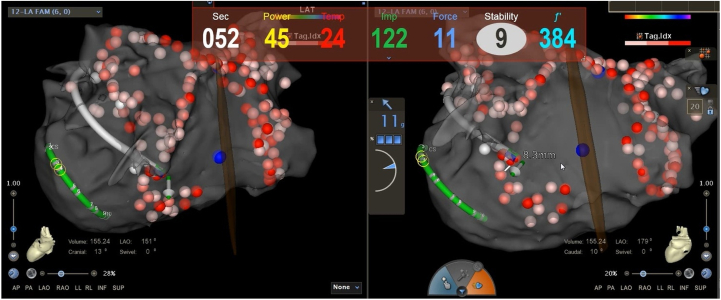
The Ablation Sites in the CARTO Map of the Left Atrium

Prior to the procedure, the patient received at least 1 month of oral anticoagulation therapy. The patient was prescribed 20 mg of rivaroxaban once daily, with the last dose taken the day before the procedure. During the procedure, 100 U/kg of unfractionated heparin was also administered. Activated clotting time was routinely monitored throughout the procedure to maintain a range of 250 to 350 seconds. The patient underwent catheter-based RFA for AF and VT under local anesthesia. The procedure was guided by the CARTO3 3D mapping system in combination with ICE. The procedure began with bilateral pulmonary vein isolation and linear ablation at the roof of the left atrium, which successfully restored sinus rhythm. This main ablation strategy of circumferential pulmonary vein isolation combined with the roof line was further augmented during the procedure. The operator identified fragmented potentials at the inferior portion of the left atrial posterior wall and, based on their experience, applied additional ablation lesions in this area to ensure comprehensive treatment (Figure 3). It took 40 minutes to perform catheter ablation for AF.

**Figure 4:**
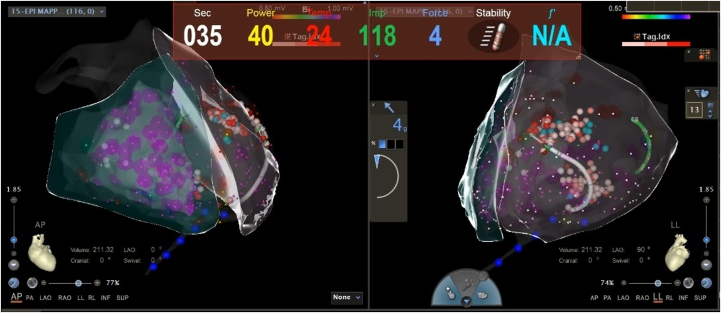
Transpericardial Epicardial Ablation

Subsequently, the RFA of VT was performed. Recurrent and frequent premature ventricular contractions, along with brief episodes of VT, occurred repeatedly prior to the operation. Surface ECG during VT onset suggested that the arrhythmia originated from the apex of the left ventricle. Epicardial mapping was initially performed prior to endocardial mapping. A 3D electroanatomic map of the epicardium was generated using the CARTO system, with data collected via an irrigated-tip RF catheter. The procedure included the use of an SmartTouch Surround Flow ablation catheter, which was introduced via the pericardial sheath, and epicardial mapping revealed a localized low-voltage area at the apex of the left ventricle with low-amplitude fragmented potentials. A power setting of 43 °C, 35 W, with saline perfusion mode was used to perform local homogenization ablation. The ablation process proceeded without complications, and subsequent isoproterenol and multiple burst stimulation tests failed to induce VT, confirming the success of the procedure (Figure 4).

## The Combination of ICE and CARTO 3D Mapping System

Using ICE combined with electroanatomic mapping (EAM) via the CARTO3 system, pericardiocentesis was successfully performed under zero fluoroscopy. The specific procedure steps were as follows:

1. Transseptal puncture (TSP): TSP was utilized during the previous RFA procedure for AF. We employ the EAM function to create a detailed reconstruction of the right atrium and both vena cava. Under ICE guidance, we carefully maneuver the mapping catheter to provide a precise reconstruction of the interatrial septum. The “tenting sign” can be visualized under ICE imaging as the puncture needle is advanced into the left atrium, completing the TSP (Figure 5).

**Figure 5:**
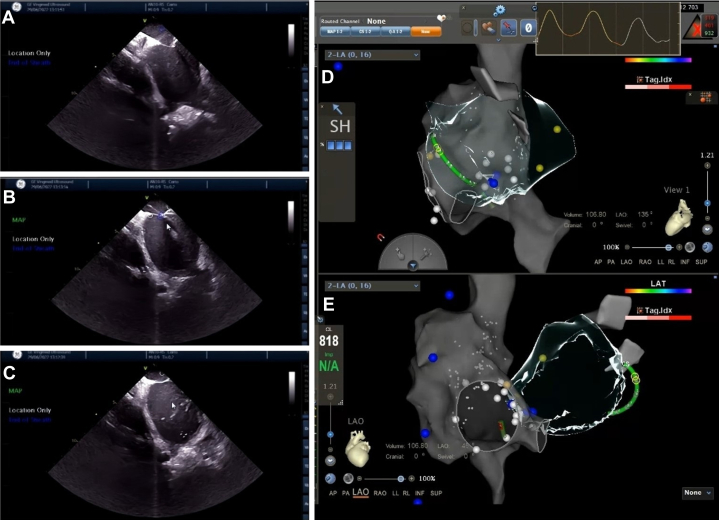
Zero-Fluoroscopy Pericardiocentesis Guided by ICE and EAM via CARTO3 System (A) ICE reveals the “tenting-like sign”; (B) transseptal puncture is executed under real-time ICE guidance; (C) the success of the puncture needle entering the left atrium is confirmed by injecting saline through the needle lumen; (D and E) EAM was used to create a detailed reconstruction of the right atrium, both venae cavae, and the interatrial septum. EAM = electroanatomic mapping; ICE = intracardiac echocardiography.
2. Pre-preparation of pericardial puncture ICE was used to trace the epicardial boundaries of the left and right ventricles, including the lateral walls, apex, and ventricular septum. These structures were mapped to their corresponding epicardial surfaces, and an epicardial anatomical model was created using CARTOSOUND (Figure 6).

**Figure 6:**
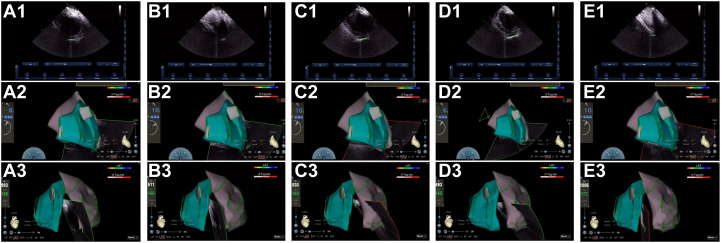
The Process of ICE Delineating the Pericardial Boundaries (A) Near-septal side of the left ventricular apex; (B) left ventricular apex; (C) left ventricular lateral wall; (D) left ventricular free wall; (E) left ventricular apex. 1) Intracavitary ultrasound plane; 2) right anterior oblique position; 3) left anterior oblique position. ICE = intracardiac echocardiography.
3. The visualization of puncture needle: Connect the unipolar lead to the distal end of the puncture needle and integrate it with the CARTO system for real-time visualization of the needle tip. A vascular-specific puncture needle (16 GA 5.25 IN/1.7 × 133 mm) was used in this case (Figure 7A). The tail end of the needle is connected to the “a” end of a custom-made connection cable (Figures 7B to 7D), which then connects the “b” and “c” ends to the PINBOX of the CARTO system. This visualization enabled accurate tracking of the puncture needle's trajectory as it advanced.

**Figure 7:**
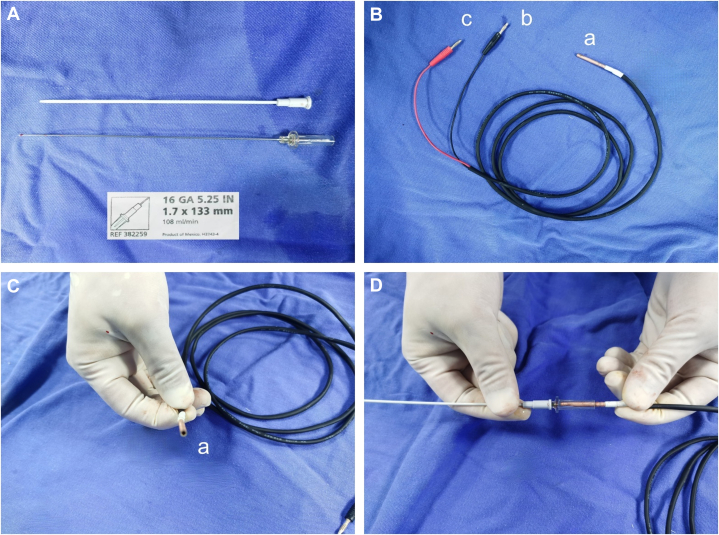
Connection of the Unipolar Lead to the Distal Tip of the Puncture Needle, Enabling Real-Time Visualization Through Integration With the CARTO Mapping System (A) The vascular-specific puncture needle, model number (16 GA 5.25 IN/1.7 × 133 mm); (B to D) the “a” end of a custom-made cable is connected to the tail end of the needle, while the “b” and “c” ends are connected to the PINBOX of the CARTO system.
4. Needle insertion: Preprocedural abdominal CT was performed to assess liver size and check for any anatomical abnormalities, including the positioning of the intestines. During the pericardiocentesis procedure, the puncture site was chosen slightly to the left of the midline, just below the xiphoid process. After the needle punctured the skin, it was directed close to the lower edge of the rib, and ICE monitoring was used throughout the procedure.

**Figure 8:**
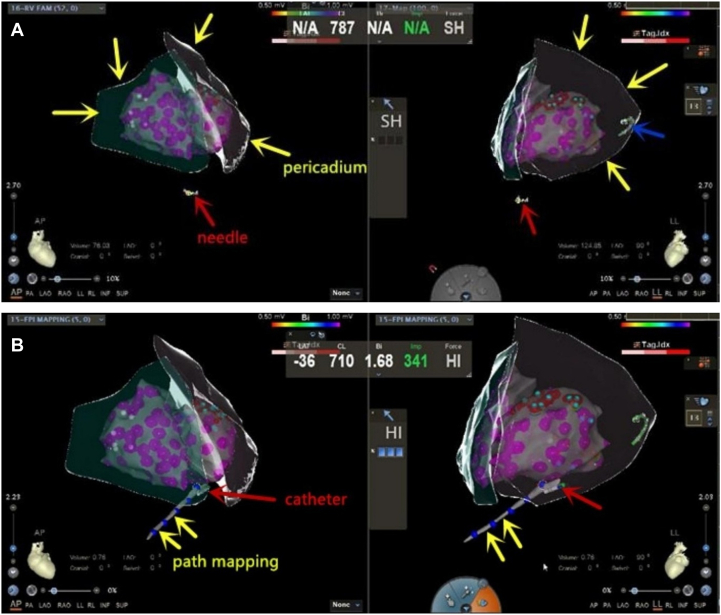
ICE/3D-Guided Pericardiocentesis and Epicardial Access Workflow (A) Under dynamic monitoring of the AP and LL projection views, the needle tip was gradually advanced to the epicardium until precise overlap was achieved. The red arrow indicates the bipolar catheter positioned by the CARTO system, representing the tip of the puncture needle; the yellow arrow denotes the epicardial anatomical model constructed using ICE combined with EAM via the CARTO3 system; and the blue arrow represents the mapping electrode located in the coronary sinus. (B) The yellow arrows delineate the trajectory of the ablation catheter as it enters the pericardial space, while the red arrows indicate the position of the ablation catheter itself within this cavity. AP = anteroposterior; ICE = intracardiac echocardiography; LL = left lateral.

The puncture needle was inserted through the conventional access point at the xiphoid process under anteroposterior and left lateral angles. As the needle tip was visualized as a monopolar catheter, it was advanced slowly under direct visualization within the 3D model, until it reached the epicardium (Figure 8A). Before pericardiocentesis, the ICE catheter is placed in the right ventricle. The catheter is then rotated and its direction adjusted to trace the structure of the parietal pericardium, helping to determine the distance and direction for the puncture needle. During epicardial ablation, the ICE remains in the right ventricle, but the positioning of the ablation mainly relies on the 3D model, rather than ICE positioning.

5. Confirmation of epicardial entry: Using a conventional pericardial puncture needle (16 GA 5.25 IN/1.7 × 133 mm), the hollow end of the needle was positioned near the epicardium. A local epicardial monopole electrogram was observed, confirming the needle's proximity to the epicardium. The breakthrough sensation was used to verify entry into the pericardial cavity.
6. Guide wire insertion: Upon entering the pericardial cavity, the puncture needle was withdrawn, leaving the outer sheath in place. A 0.035-inch guide wire was then advanced to 150 cm, with a bipolar guide catheter tail attached to the wire's end for visualization in the 3D model. The guide wire was smoothly introduced into the pericardial cavity through the sheath, with its motion trajectory used to confirm successful entry into the pericardium.
7. Establishing pericardial access: An 8F venous sheath was introduced through the pericardial access point to establish a pathway into the pericardium, completing the pericardiocentesis procedure (Figure 8B).

The catheter ablation for VT involved 30 minutes of endocardial ablation and 40 minutes of epicardial ablation.

## Follow-Up

The patient has not experienced any recurrence of palpitations or chest tightness postoperatively. He has undergone regular follow-up visits at our outpatient department 1 and 2 years after the procedure, with no evidence of recurrent AF or VT.

## Discussion

The left ventricular myocardium's substantial thickness, often exceeding 10 mm, poses a significant challenge for traditional RFA energy to penetrate effectively. Notably, some VT originate from the epicardium,^1^ rendering endocardial surface ablation insufficient and necessitating epicardial approaches. Since Sosa et al^2^ pioneered epicardial mapping and ablation in 1996, these techniques have evolved into crucial modalities for treating VT.^3^

Pericardiocentesis is fundamental to and a prerequisite for epicardial ablation. Conventionally, this procedure is performed under X-ray guidance, where the puncture needle's trajectory and contrast medium imaging is used to ascertain entry into the pericardial space. However, the pericardium typically contains only 15 to 25 mL of physiological serous fluid,^4^ with the parietal and visceral layers in close apposition, making precise localization of the pericardial cavity challenging under fluoroscopy alone. The study reports a 5% to 10% incidence of complications associated with epicardial puncture, mapping, and ablation procedures.^5^ In severe cases, these complications can be life-threatening and may necessitate thoracotomy. This underscores the importance of refining techniques and developing more precise approaches to epicardial interventions in electrophysiology.

This study demonstrates a refined pericardiocentesis approach that significantly enhances the safety and precision of epicardial access. In our case, the integration of ICE with the CARTO 3D EAM system allowed for real-time, high-resolution visualization of cardiac and pericardial anatomy. Key innovations included preprocedural ICE-based anatomical modeling of the epicardial boundaries of the left and right ventricles, including the lateral walls, apex, and ventricular septum, as well as dynamic visualization of the puncture needle by connecting a unipolar lead to its distal tip. This enabled precise localization of the needle tip within the 3D mapping field, facilitating accurate navigation toward the pericardial space under full visualization. Furthermore, the incorporation of a visualized guidewire system, represented as a bipolar catheter within the mapping model, provided an additional layer of safety by confirming the guidewire's trajectory and final positioning within the pericardial cavity. These innovations collectively improved the overall safety, reproducibility, and operator confidence in performing pericardiocentesis, especially in anatomically challenging or high-risk cases.

Compared to conventional fluoroscopy-guided catheter ablation, the zero-fluoroscopy approach using ICE integrated with 3D EAM systems such as CARTO offers several notable advantages. First and foremost, the elimination of fluoroscopy significantly reduces radiation exposure for both patients and medical staff, addressing long-term occupational health risks including malignancies, cataracts, and orthopedic strain from lead shielding. This is particularly beneficial in younger patients, women of childbearing age, and high-volume electrophysiology teams. In terms of procedural efficacy, ICE allows for real-time visualization of cardiac anatomy and catheter-tissue contact, enhancing precision during TSP and epicardial access. The anatomical fidelity provided by ICE and 3D mapping improves targeting of arrhythmogenic substrates, particularly in structurally complex hearts or in cases requiring epicardial ablation.

Furthermore, in scenarios where AF ablation is not indicated, a fully fluoroless strategy remains achievable. The combination of ICE and EAM alone allows for high-resolution endocardial activation and substrate mapping, which can be completed without the need for fluoroscopy. If, following thorough endocardial ablation, VT persists and an epicardial origin is suspected, pericardial access can be performed using the same ICE- and EAM-guided technique described in this report. Under real-time ICE imaging and with precise visualization of the puncture needle and guidewire within the CARTO 3D mapping system, safe and accurate epicardial access can be established entirely without radiation exposure. The minimum required setup includes an ICE catheter, a 3D mapping system, a unipolar-visualized puncture needle, and a bipolar guidewire—all of which enable full procedural guidance from access to ablation without fluoroscopic assistance. This approach provides a viable zero-fluoroscopy solution even in cases limited to VT ablation, further expanding the clinical applicability of radiation-free strategies.

However, the zero-fluoroscopy technique is not without limitations. It requires substantial operator experience in ICE interpretation and navigation within 3D mapping systems and may be associated with a longer learning curve. Moreover, in cases of unexpected anatomical variants or complications such as pericardial tamponade, the lack of fluoroscopic backup can pose challenges in rapid visualization and management. There is also limited widespread availability of ICE in some regions, and its cost may be prohibitive in lower-resource settings.

## Funding Support and Author Disclosures

This case report was approved by the Research Ethics Committee of Suzhou Kowloon Hospital, Shanghai Jiao Tong University School of Medicine, China. Informed consent was obtained from the patient. The authors have reported that they have no relationships relevant to the contents of this paper to disclose.
